# Expanding the Clinical Spectrum of Bardet-Biedl Syndrome: Chronic Liver Disease in an Adult Patient

**DOI:** 10.7759/cureus.103180

**Published:** 2026-02-07

**Authors:** Ramya M.R., Swathi B.S., Madhurya M., Sinchana T.P.

**Affiliations:** 1 General Medicine, JSS Medical College, Mysore, IND

**Keywords:** bardet-beidel syndrome, chronic liver disease, hypogonadotrophic hypogonadism, post-axial polydactyly, retinitis pigmentosa (rp)

## Abstract

Bardet-Biedl syndrome (BBS) is a rare autosomal recessive ciliopathy with multisystem involvement, classically characterized by retinal dystrophy, obesity, postaxial polydactyly, and renal abnormalities. We report a 30-year-old patient who presented with progressive visual impairment, truncal obesity, and postaxial polydactyly. Comprehensive evaluation revealed clinical, biochemical, and radiological features consistent with chronic liver disease, with common etiologies - including viral hepatitis, alcohol-related liver disease, and autoimmune disorders - excluded. Recognition of the constellation of systemic features led to the diagnosis of BBS in adulthood, highlighting a significant delay in syndromic identification. This case emphasizes chronic liver disease as a potentially underrecognized manifestation of BBS and illustrates the impact of delayed diagnosis on disease burden. Early recognition of BBS and systematic multisystem screening, including hepatic evaluation, is essential to enable timely intervention and improve long-term outcomes.

## Introduction

Bardet-Biedl syndrome (BBS) is a rare autosomal recessive ciliopathy characterized by marked phenotypic heterogeneity and multisystem involvement. The cardinal clinical features include retinal dystrophy, truncal obesity, postaxial polydactyly, renal abnormalities, learning difficulties, and hypogonadism, reflecting the widespread impact of primary ciliary dysfunction on organ development and homeostasis [1]. The estimated global prevalence of BBS ranges from approximately one in 100,000 to one in 160,000 in the general population, with significantly higher prevalence reported in certain populations due to founder effects and consanguinity [1,2].

In India, the true incidence and prevalence of BBS remain poorly defined, with available data largely restricted to isolated case reports and small case series. This likely reflects substantial underdiagnosis and delayed recognition, attributable to phenotypic variability, limited awareness, and the absence of routine genetic testing in many clinical settings [3]. As a result, the diagnosis is frequently established only in adolescence or adulthood, often after the development of irreversible organ involvement.

While renal and metabolic complications are well-recognized contributors to morbidity and mortality in BBS, hepatic involvement has historically received less attention. Emerging evidence suggests that liver abnormalities - ranging from hepatic steatosis to congenital hepatic fibrosis, portal hypertension, and cirrhosis - constitute an important but underrecognized component of the expanding phenotypic spectrum of BBS [4,5]. The typically indolent course of hepatic disease and lack of routine surveillance frequently result in delayed detection, with liver involvement identified only after the onset of clinically significant complications. This case highlights the need for increased awareness of hepatic manifestations in BBS and supports the inclusion of systematic liver evaluation in affected individuals.

## Case presentation

A 30-year-old male from Mysore, Karnataka, India, was admitted to the general medicine ward with complaints of progressive bilateral lower-limb swelling, abdominal distension, and reduced urine output for one week. He also reported a history of generalized weakness and decreased appetite. There was no history of alcohol consumption, jaundice, hematemesis, melena, or known chronic liver disease before this admission.

**Figure 1:**
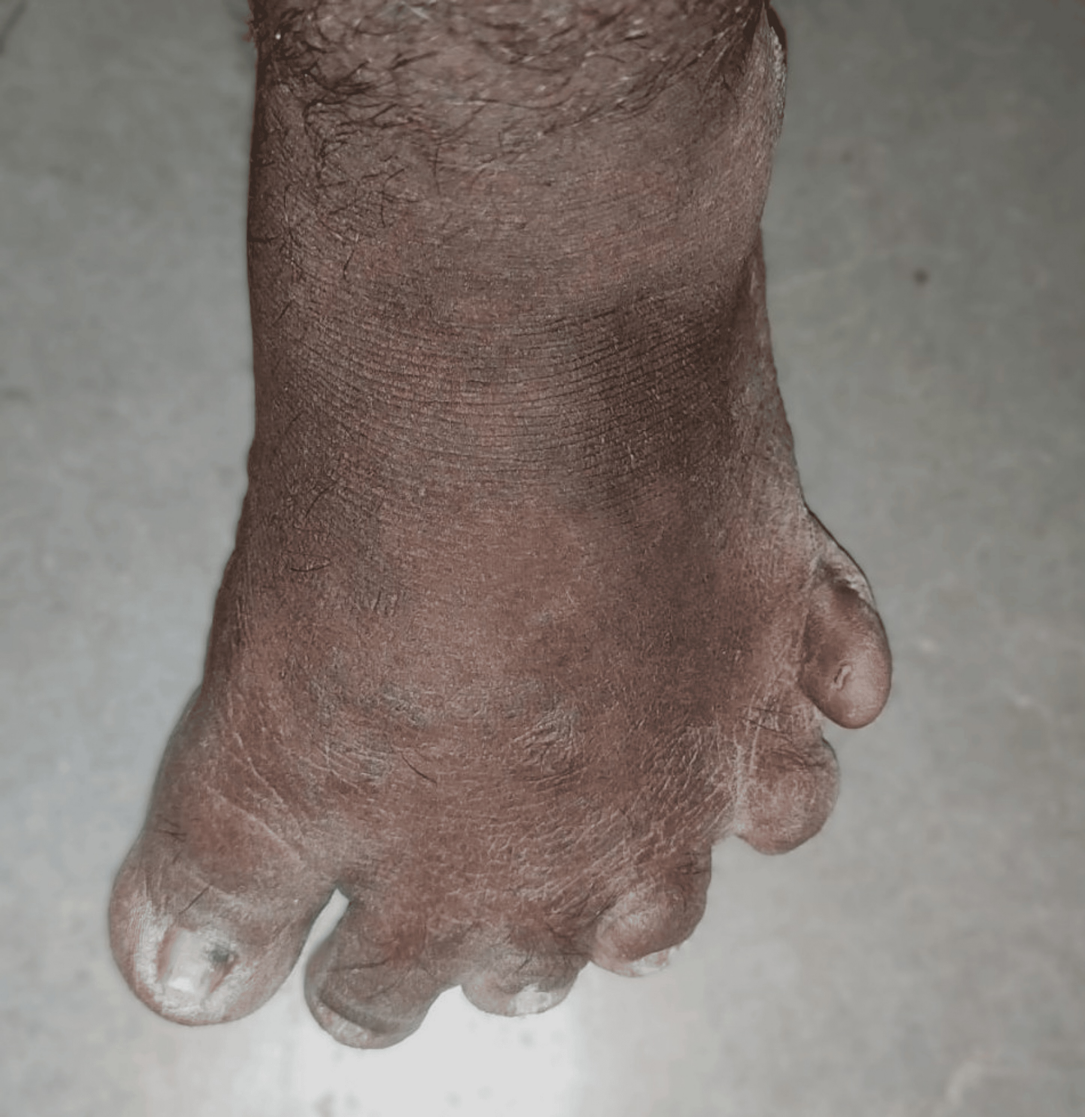
Short toes and polydactyly

On further history, the patient reported long-standing visual impairment since childhood and obesity from early adolescence. There was a history suggestive of postaxial brachydactyly and polydactyly involving the lower limbs, as shown in Figure 1, which had not been previously investigated. There was no significant family history of similar illness. There was no history of diabetes mellitus, hypertension, tuberculosis, or epilepsy.

**Figure 2:**
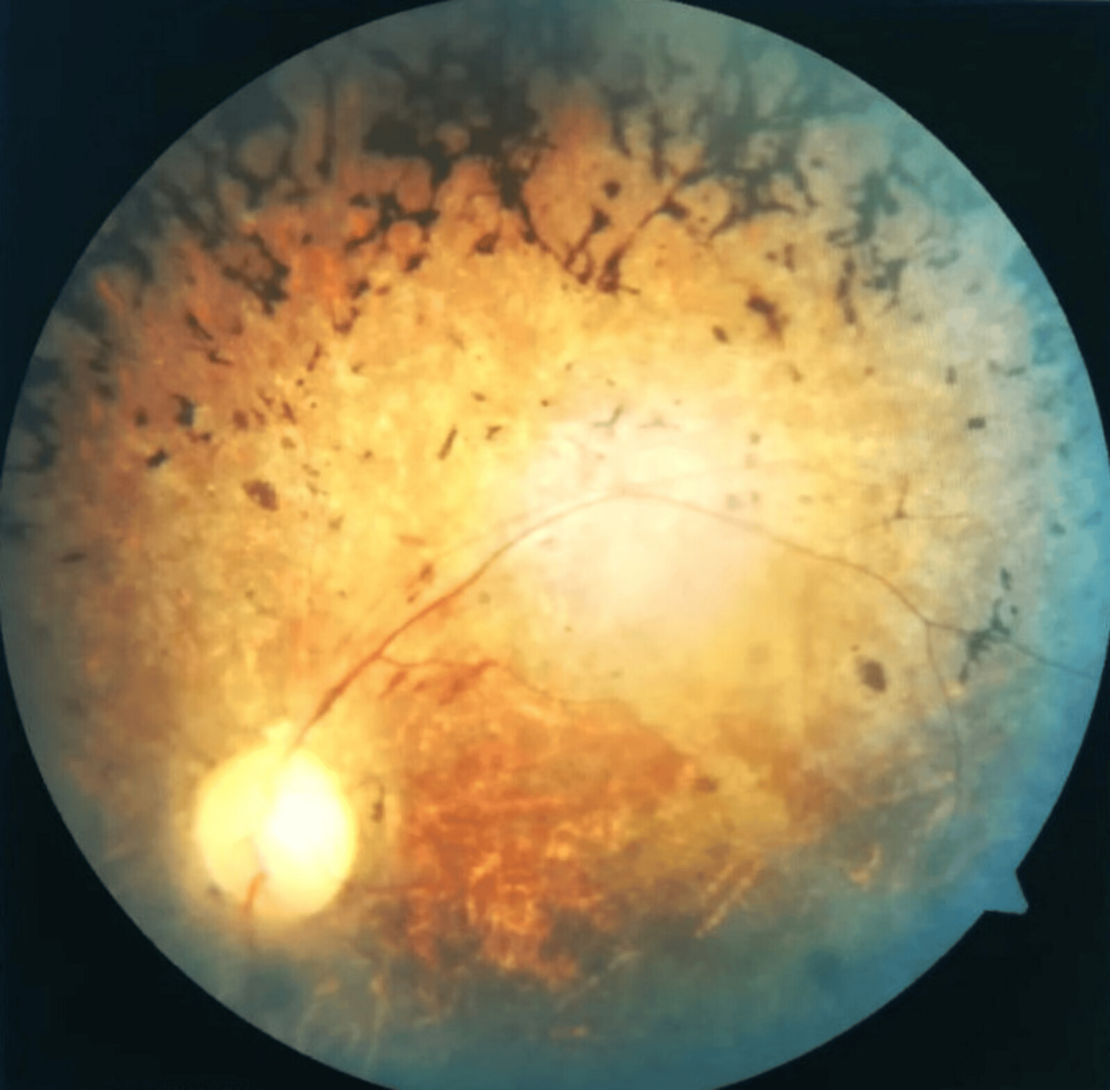
Bone-spicule–shaped pigmentary deposits, attenuation of retinal arterioles, and waxy pallor of the optic disc, features suggestive of retinitis pigmentosa

On examination, the patient was obese (body mass index: 35.05), with a body habitus suggestive of truncal obesity. He was conscious and oriented. Pallor and bilateral pitting pedal edema were present. Vital signs were within normal limits. Abdominal examination revealed distension with shifting dullness, suggestive of ascites. Cardiovascular and respiratory system examinations were unremarkable. Intellectual functioning was assessed using the Wechsler Adult Intelligence Scale (WAIS), which demonstrated below-average cognitive performance consistent with mild intellectual impairment, with relative deficits in abstract reasoning and problem-solving as well as preservation of basic adaptive functioning. Ophthalmological examination revealed reduced visual acuity bilaterally. Fundoscopic image in Figure 2 shows bilateral mid-peripheral bone-spicule-shaped pigmentary deposits, attenuation of retinal arterioles, and waxy pallor of the optic disc, features consistent with retinal dystrophy.

Baseline investigations revealed anemia and thrombocytopenia. Liver function tests demonstrated hypoalbuminemia with mild elevation of transaminases and alkaline phosphatase, consistent with chronic liver disease (Table 1). Renal function tests showed elevated serum urea and creatinine, suggestive of acute kidney injury in the setting of chronic liver disease. Urine exam was normal. Viral markers for hepatitis B and C were negative. Serum ferritin and ceruloplasmin levels were normal. Autoimmune liver disease workup was also negative. Ascitic fluid analysis showed a transudative picture with low protein content and no evidence of malignant cells. Ascitic fluid adenosine deaminase levels were within normal limits, excluding tubercular peritonitis. The presence of Child-Pugh class B cirrhosis with a Model for End-Stage Liver Disease (MELD) score of 16 indicates moderate severity of chronic liver disease at presentation.

**Table 1:** Liver function test and coagulation parameters

Parameters	Observed Value	Reference Range
Total bilirubin	1.07 mg/dL	0.2-1.2 mg/dL
Direct (conjugated) bilirubin	0.72 mg/dL	0.0-0.3 mg/dL
Aspartate aminotransferase (AST)	38.9 U/L	5-35 U/L
Alanine aminotransferase (ALT)	43.0 U/L	5-35 U/L
Serum albumin	2.73 g/dL	3.5-5.0 g/dL
Serum globulin	3.7 g/dL	2.0-3.5 g/dL
Total protein	6.43 g/dL	6.0-8.0 g/dL
Alkaline phosphatase (ALP)	178 IU/L	44-147 IU/L
Prothrombin time (PT)	16	11-13.5 seconds
International normalized ratio (INR)	1.18	0.8-1.2

Glycemic evaluation revealed elevated HbA1c (7.5%), consistent with diabetes mellitus. Thyroid function tests showed hypothyroidism. Hormonal evaluation revealed low serum testosterone with inappropriately normal gonadotropins, consistent with hypogonadotropic hypogonadism (Table 2).

**Table 2:** Hormonal and endocrine profile

Test	Result	Reference Range
HbA1c (%)	7.5	<6.5
Thyroid-stimulating hormone (TSH) (µIU/mL)	49	0.4-4.2
Serum testosterone (ng/dL)	65.2	241-827
Follicle-stimulating hormone (FSH) (mIU/mL)	12.83	1-14
Luteinizing hormone (LH) (mIU/mL)	6.33	0.7-7.4

Ultrasonography of the abdomen revealed coarse hepatic echotexture with irregular margins, splenomegaly, and moderate ascites, consistent with chronic parenchymal liver disease. The portal vein was of normal caliber with preserved flow. Both kidneys were normal in size and echotexture. Upper gastrointestinal endoscopy revealed Grade B esophagitis with esophageal varices and features of portal hypertensive gastropathy, confirming portal hypertension.

The patient was managed conservatively with salt restriction, diuretics, albumin infusion, non-selective beta-blockers, insulin, thyroid hormone replacement, and supportive care. Ascites and pedal edema improved with treatment. The patient was discharged in a stable condition with advice for regular follow-up.

## Discussion

The diagnosis of BBS in this patient was established using clinical diagnostic criteria, which remain the cornerstone of diagnosis in the absence of genetic confirmation. According to the criteria proposed by Beales et al., the presence of four primary features or three primary features with two secondary features is sufficient for diagnosis [1]. In the present case, the patient fulfilled multiple primary and secondary criteria, as outlined in Table 3, thereby meeting the threshold for a clinical diagnosis of BBS.

**Table 3:** Diagnostic criteria for Bardet-Biedl syndrome fulfilled as per Beales et al. [1]

Primary Features (Beales et al.)	Present /Absent (+/−)	Secondary Features (Beales et al.)	Present/Absent (+/−)
Rod–cone dystrophy	+	Speech delay	−
Postaxial polydactyly	+	Developmental delay	+
Obesity	+	Diabetes mellitus	+
Learning difficulties	+	Dental anomalies	−
Hypogonadism (male)	+	Congenital heart disease	−
Renal anomalies	−	Brachydactyly/syndactyly	+
		Ataxia/poor coordination	−
		Anosmia/hyposmia	−

Genetic testing was not performed due to limited in-house availability of comprehensive molecular diagnostics at our tertiary care center in Mysore. Access to next-generation sequencing panels requires referral to external private laboratories and is associated with significant out-of-pocket costs. In this case, financial constraints precluded external testing. Additionally, the patient fulfilled established clinical diagnostic criteria for BBS, and genetic confirmation was unlikely to alter immediate management. This reflects common real-world limitations in resource-constrained settings in India, where access to affordable genetic testing remains limited.

Ophthalmologic involvement and pathophysiology

Ophthalmologic manifestations are among the most consistent and diagnostically significant features of BBS. Retinal dystrophy, typically presenting as rod-cone dystrophy or retinitis pigmentosa, arises from primary ciliary dysfunction in photoreceptor cells. Disruption of the BBSome complex impairs intraflagellar transport, leading to progressive photoreceptor degeneration [3,6]. As retinal dystrophy is nearly universal, ophthalmologic evaluation serves as a critical diagnostic anchor in patients with multisystem disease [1,2].

Skeletal deformities

Skeletal abnormalities form an important component of the phenotypic spectrum of BBS and are included among the secondary diagnostic criteria. Common manifestations include postaxial polydactyly, brachydactyly, and, less commonly, syndactyly [1,2]. These deformities result from defective primary ciliary signaling during embryogenesis, particularly disruption of the Sonic Hedgehog (SHH) signaling pathway, which plays a crucial role in limb bud patterning and digit specification [6].

Impaired ciliary function in mesenchymal and chondrocyte precursors leads to abnormal skeletal morphogenesis, a mechanism shared across ciliopathies and mediated by disrupted intraflagellar transport and centrosomal signaling [6].

Endocrine and metabolic involvement

Endocrine dysfunction contributes substantially to morbidity in BBS. Diabetes mellitus is increasingly recognized as part of the metabolic phenotype and is attributed to a combination of truncal obesity, insulin resistance, and hypothalamic dysfunction related to ciliary abnormalities [2,7]. Population-based studies have demonstrated a higher prevalence of impaired glucose tolerance and early-onset type 2 diabetes mellitus in individuals with BBS compared with the general population [8]. Recent clinic-based cohorts further confirm the high burden of metabolic and endocrine abnormalities in affected individuals [9]. These metabolic disturbances may influence the severity of involvement in other organ systems.

Hypogonadotropic hypogonadism is a well-established endocrine manifestation of BBS and results from impaired hypothalamic-pituitary signaling [1,2,9]. Beyond reproductive implications, this abnormality affects bone mineral density, body composition, and psychosocial well-being, underscoring the need for comprehensive hormonal evaluation.

Hepatic involvement

Hepatic involvement, although not part of the classical diagnostic criteria for BBS, is increasingly recognized as a significant manifestation within its expanding phenotypic spectrum [4,5]. In the present case, liver disease constituted the primary reason for hospital admission and represented a clinically advanced yet previously unrecognized complication of BBS.

The patient presented with progressive ascites, bilateral pedal edema, hypoalbuminemia, thrombocytopenia, and esophageal varices, consistent with portal hypertension secondary to chronic liver disease. There was no history of alcohol consumption, viral hepatitis, or known chronic liver disease, and investigations for viral hepatitis and autoimmune liver disease were negative. Normal serum ferritin and ceruloplasmin levels further excluded secondary causes of chronic liver disease. These findings strongly suggest that hepatic pathology represents an intrinsic manifestation of the underlying ciliopathy rather than a coincidental condition [4].

Hepatic involvement in BBS has been described as a heterogeneous spectrum, ranging from hepatic steatosis to advanced fibrosis and portal hypertension, often with delayed clinical recognition [5]. The clinical course in this patient mirrors these observations, with portal hypertension becoming evident before overt hepatic decompensation.

The most characteristic hepatic pathology associated with BBS is congenital hepatic fibrosis, a fibropolycystic liver disease resulting from ductal plate malformation [4]. Although histological confirmation was not obtained, the constellation of portal hypertension, preserved portal venous flow, mild transaminase elevation, and absence of significant hepatocellular failure is highly suggestive of this entity. Such patients may retain near-normal liver enzyme levels despite advanced portal hypertension, contributing to underdiagnosis [4].

The pathogenesis of hepatic involvement in BBS is closely linked to primary ciliary dysfunction in cholangiocytes, which disrupts bile duct development and signaling, leading to abnormal ductal plate remodeling and progressive periportal fibrosis [4,10]. This mechanism supports hepatic disease as a direct manifestation of the underlying genetic defect rather than a secondary phenomenon.

Metabolic abnormalities commonly associated with BBS, including truncal obesity, diabetes mellitus, and hypogonadotropic hypogonadism, may further accelerate hepatic fibrogenesis and disease progression [7-9]. Management of hepatic disease in BBS remains supportive and complication-based, as no disease-specific therapies are currently available [4,5]. Long-term prognosis depends on vigilant surveillance for portal-hypertension-related complications and optimization of metabolic comorbidities.

## Conclusions

This case expands the recognized clinical spectrum of BBS by illustrating the relevance of hepatic involvement alongside established systemic manifestations. It reinforces the need for a structured, multisystem evaluation guided by clinical diagnostic criteria and highlights the value of integrated care in managing this complex ciliopathy.

## References

[1] Beales PL, Elcioglu N, Woolf AS, Parker D, Flinter FA (1999). New criteria for improved diagnosis of Bardet-Biedl syndrome: results of a population survey. J Med Genet.

[2] Forsythe E, Beales PL (2013). Bardet-Biedl syndrome. Eur J Hum Genet.

[3] Mockel A, Perdomo Y, Stutzmann F, Letsch J, Marion V, Dollfus H (2011). Retinal dystrophy in Bardet-Biedl syndrome and related syndromic ciliopathies. Prog Retin Eye Res.

[4] Gunay-Aygun M (2009). Liver and kidney disease in ciliopathies. Am J Med Genet C Semin Med Genet.

[5] Day LB, Quammie C, Héon E, Bhan A, Batmanabane V, Dai T, Kamath BM (2016). Liver anomalies as a phenotype parameter of Bardet-Biedl syndrome. Clin Genet.

[6] Gerdes JM, Davis EE, Katsanis N (2009). The vertebrate primary cilium in development, homeostasis, and disease. Cell.

[7] Feuillan PP, Ng D, Han JC (2011). Patients with Bardet-Biedl syndrome have hyperleptinemia suggestive of leptin resistance. J Clin Endocrinol Metab.

[8] Moore SJ, Green JS, Fan Y (2005). Clinical and genetic epidemiology of Bardet-Biedl syndrome in Newfoundland: a 22-year prospective, population-based, cohort study. Am J Med Genet A.

[9] Mujahid S, Hunt KF, Cheah YS (2018). The endocrine and metabolic characteristics of a large Bardet-Biedl syndrome clinic population. J Clin Endocrinol Metab.

[10] Masyuk AI, Masyuk TV, LaRusso NF (2008). Cholangiocyte primary cilia in liver health and disease. Dev Dyn.

